# A novel plasma circular RNA circFARSA is a potential biomarker for non‐small cell lung cancer

**DOI:** 10.1002/cam4.1514

**Published:** 2018-05-02

**Authors:** Dong Hang, Jing Zhou, Na Qin, Wen Zhou, Hongxia Ma, Guangfu Jin, Zhibin Hu, Juncheng Dai, Hongbing Shen

**Affiliations:** ^1^ Department of Epidemiology and Biostatistics School of Public Health Nanjing Medical University Nanjing 211166 China; ^2^ Jiangsu Key Lab of Cancer Biomarkers, Prevention and Treatment Collaborative Innovation Center for Cancer Medicine Nanjing Medical University Nanjing 211166 China

**Keywords:** Circular RNA, lung cancer, plasma, RNA sequencing

## Abstract

Emerging evidence indicates that circular RNAs (circRNAs) are implicated in cancer development. This study aimed to evaluate whether circulating circRNAs may serve as novel biomarkers for non‐small cell lung cancer (NSCLC). We used RNA sequencing (RNA‐seq) and quantitative real‐time PCR to explore cancer‐related circRNAs. Bioinformatics and functional analyses were performed to reveal biological effects of circRNAs on lung cancer cells. A total of 5471 distinct circRNAs were identified by total RNA‐seq, in which 185 were differentially expressed between cancerous and adjacent normal tissues. A circRNA derived from exon 5–7 of the *FARSA* gene, termed circFARSA, was observed to increase in cancerous tissues (*P *=* *0.016), and was more abundant in patients’ plasma than controls (*P *<* *0.001). Overexpression of circFARSA in A549 cell line significantly promoted cell migration and invasion. In silico analysis suggested that circFARSA might sponge miR‐330‐5p and miR‐326, thereby relieving their inhibitory effects on oncogene fatty acid synthase. Summarily, this study revealed circRNA profile of NSCLC for the first time and provided the evidence of plasma circFARSA as a potential noninvasive biomarker for this malignancy.

## Introduction

Lung cancer is the most common and deadly malignancy worldwide, with more than 1.8 million new cases and nearly 1.6 million deaths estimated in 2012 1. Among these cases, non‐small cell lung cancer (NSCLC) patients account for 80%, and their 5‐year survival rate remains only 15% 2. Identification of individuals with early‐stage lung cancer has the potential to reduce the mortality, as demonstrated by the National Lung Cancer Screening Trial based on annual low‐dose computerized tomographic screening 3. However, 96% of the pulmonary abnormalities observed in that trial were benign lesions, and periodic radiological exposure may cause harmful effects 3. Therefore, developing specific and noninvasive (e.g., blood‐based) biomarker assay is crucial to improve the identification of individuals at high risk for lung cancer.

Circular RNAs (circRNAs) were regarded as the by‐products of splicing or splicing errors with low abundance until recently 4, 5. With rapid advances in high‐throughput sequencing and bioinformatics technologies, abundant circRNAs have been discovered 6, 7, 8. CircRNAs can regulate gene expression through interactions with microRNAs (miRNAs) or other competing endogenous RNAs and participate in various biological activities 9, 10. Increasing evidence suggests that aberrant circRNA expression may contribute to the development of different cancers, such as lung cancer 11, 12, esophageal squamous cell carcinoma 13, hepatocellular cancer 14, and colorectal cancer 15.

Compared to liner RNAs that terminate in 5′ caps and 3′ tails, circRNAs form covalently closed loops without 5′ or 3′ polarities and have been confirmed relatively stable and resistant to RNase R 16. Notably, a recent study reported that circRNAs originated from cancer tissues could enter the circulation and be measured in the serum 17. Therefore, circulating circRNAs research may open up a new field for molecular diagnosis of cancer.

This study aimed to assess the potential value of plasma circRNAs as novel biomarkers for NSCLC. We first performed high‐throughput RNA sequencing (RNA‐seq) to identify differentially expressed circRNAs in 10 paired NSCLC tissues and adjacent normal counterparts. A circRNA derived from exon 5–7 of the *FARSA* gene, termed circFARSA, was found to be significantly upregulated in cancerous tissues and abundant in corresponding plasma. We further determined circFARSA levels in plasma from 50 patients with NSCLC and 50 healthy controls using qRT‐PCR. Bioinformatics and functional analyses were also performed to show biological clues of cirFARSA in lung cancer.

## Material and Methods

### Study population and sample collection

This study was approved by the institutional review board of Nanjing Medical University and conformed to the Declaration of Helsinki. A two‐stage design was performed (Fig. S1). In the screening stage, 10 pairs of cancerous and adjacent normal tissues used for total RNA‐seq were collected from NSCLC patients attending Affiliated Cancer Hospital of Nanjing Medical University (Nanjing, Jiangsu Province, China) between October 2014 and December 2015 (Table S1). Tissue specimens were preserved in RNAlater solution, and HE‐stained tissue sections were examined to ensure representative sampling. In the validation stage, fifty patients with NSCLC were recruited from the same hospital and provided plasma specimens. The exclusion criteria were having a prior history of other cancers, metastatic cancer from other sites, or having received chemotherapy or radiotherapy. Fifty controls were randomly selected from a cohort of periodic health examination participants in the hospital during the same time. The controls had no cancer and were individually matched to cases by age (±5 years) and gender. After signing an informed consent, each participant was interviewed face‐to‐face by a trained interviewer to collect information on demographic characteristics and lifestyles, such as age, sex, and smoking.

### Total RNA‐seq and circRNA identification

Total tissue RNA was extracted using RNeasy Mini Kit (Qiagen, Hilden, Germany) and was analyzed by an Agilent 2100 Bioanalyzer system (Agilent Biotechnologies, Palo Alto, CA) with the RNA 6000 Nano Labchip Kit. Only samples of high‐quality RNA (RNA Integrity Number ≥7.5) were used in the subsequent construction of RNA‐seq libraries. Ribosome‐minus RNA was fractionated from total RNA samples, and RNA‐seq libraries were constructed orderly by RNA fragmentation, random hexamer‐primed cDNA synthesis, linker ligation, and PCR amplification using a TruSeq RNA Sample Prep Kit (Illumina, San Diego, CA). The purified cDNA libraries were sequenced on the Illumina HiSeq1500 platform (paired‐end, 100‐bp read).

Illumina BCL files were converted to FASTQ format using CASAVA v1.8.2. Initial quality controls were conducted by Trimmomatic version 0.32.43 to trim off adapters and low quality bases (ILLUMINACLIP: adapter.fa:2:30:10 LEADING:3 TRAILING:3 SLIDINGWINDOW:4:15 MINLEN:20). Qualified reads were sent to CIRCexplorer v 1.1.10 pipeline to identify and quantify circRNAs with default parameters 18. A circRNA was called with the support of at least two unique back‐spliced reads. The total number of reads that spanned back‐spliced junctions was used as an absolute measure of circRNA abundance.

### Plasma RNA extraction and cDNA synthesis

Total plasma RNA was extracted using TRIzol LS Reagent (Invitrogen, Carlsbad, CA) and RNeasy Plus Mini Kit (Qiagen), according to the manufacturer's instructions. As an internal control, 100 ng total RNA of C. elegans was artificially added into each plasma specimen (200 μL) before RNA extraction. The purity and concentration of RNA samples were determined with the NanoDrop ND‐2000 (Thermo Fisher Scientific, Wilmington, DE). The integrity of RNA was assessed by agarose gel electrophoresis. Template cDNA was synthesized by reverse transcription using random primers (PrimeScript RT Master Mix, Takara, Japan).

### qRT‐PCR assay

The qRT‐PCR was performed by using the SYBR Premix Ex Taq^™^ II (Taraka, Japan) on ABI 7900 system. Cel‐circRNA 9 (cel_9), which is highly expressed in C. elegans and RNase R resistant 6, was selected as an internal reference. Divergent primers of circFARSA and cel‐9 were designed as follows: 5′‐GCTCCTTCTGGAACTTTGAC‐3′ (sense) and 5′‐TTGCTCACCCAGTAGGTCTT‐3′ (antisense) for circFARSA; 5′‐TTGCAGCTCTCATAGAAGGAACCG‐3′ (sense) and 5′‐GTTTCAGCCGAGACTAGACTTTGAGC‐3′ (antisense) for cel_9. Representative PCR products were sequenced to confirm the back‐splice junctions of circRNAs. The data of qRT‐PCR were analyzed by the ΔCt method, and 2^−ΔCt^ represented a relative expression level of circRNAs. The assay was performed by triplicate in three independent experiments to guarantee reliability.

### Plasmid construction

The cDNA encoding circFARSA in SH‐SY5Y cells was PCR‐amplified by using primers 5′‐CGGAATTCTGAAATATGCTATCTTACAGGACTCTGAAGACCTACTGGGTGAG‐3′and 5′‐CGGGATCCTCAAGAAAAAATATATTCACCTCGAAGGAAGAAGGTGTCGTGCT‐3′. The target fragment of 394‐bp orderly contained EcoR I site, splice acceptor AG, circFARSA sequence, splice donor GT, and Bam HI site. After digestion with EcoR I and Bam HI enzymes, the fragment was cloned into the pLCDH‐ciR vector (Guangzhou Geneseed Biotech Co, Guangzhou, China) for the circulation of target transcripts 19. The constructed plasmid was verified by direct Sanger sequencing and qRT‐PCR.

### Cell proliferation, migration, and invasion assays

A human lung cancer cell line, A549, was purchased from ATCC (American Type Culture Collection) and confirmed by short tandem repeat (STR) profiling. Proliferation assays were performed by using CCK‐8 kit (Doindo, Japan). At 0, 12, 24, 36, 48, and 72 h, the CCK‐8 reagent was added into each well and incubated at 37°C for 2 h. The optical density at 450 nm was measured by an automatic microplate reader (BioTek, Winooski, VT). Cell migration and invasion were assessed by using Costar Transwell plates (Coring, NY), as described previously 20. All experiments were repeated independently three times.

### In silico analysis

CircRNA sequence was treated as a seed to analyze the circRNA–miRNA interaction by using Circular RNA Interactome based on the TargetScan algorithm 21, 22. In addition, miRNA–mRNA interaction and KEGG analyses were conducted with DIANA‐TarBase v.7.0, which incorporated experimentally supported data from hundreds of publications and more than 150 CLIP‐Seq libraries 23. The interaction network of circRNA–miRNA–mRNA was constructed by Cytoscape software (http://www.cytoscape.org/).

RNA‐seq data (level 3) for 488 LUAD (including 57 paired cancerous and adjacent normal tissues) and 489 LUSC patients (including 50 paired cancerous and adjacent normal tissues) were downloaded from the TCGA data portal (https://portal.gdc.cancer.gov/).

The prognostic value of the FASN was analyzed by a web‐based Kaplan–Meier plotter with multiple microarray data (http://www.kmplot.com/lung). Jetset scoring was used to identify the optimal microarray probe set for FASN 24.

### Statistical analysis

Statistical analysis was performed by GraphPad Prism 6 (GraphPad Software, San Diego, CA) and R software version3.3.0 (Free Software Foundation's GNU project). DESeq2 package in Bioconductor was used to identify differentially expressed circRNAs in RNA‐seq data. Hierarchical clustering analysis was performed using the most significant differentially expressed circRNAs by Cluster and TreeView programs. We assessed the relationship of circRNAs between tissues and plasma by calculating Pearson correlation coefficient. Differences in plasma circRNAs between cases and controls were assessed by paired t‐tests after log‐normal transformation of raw expression data. *P *<* *0.05 was considered statistically significant, and all tests were two‐tailed.

## Results

### Identification of differentially expressed circRNA profile

**Figure 1:**
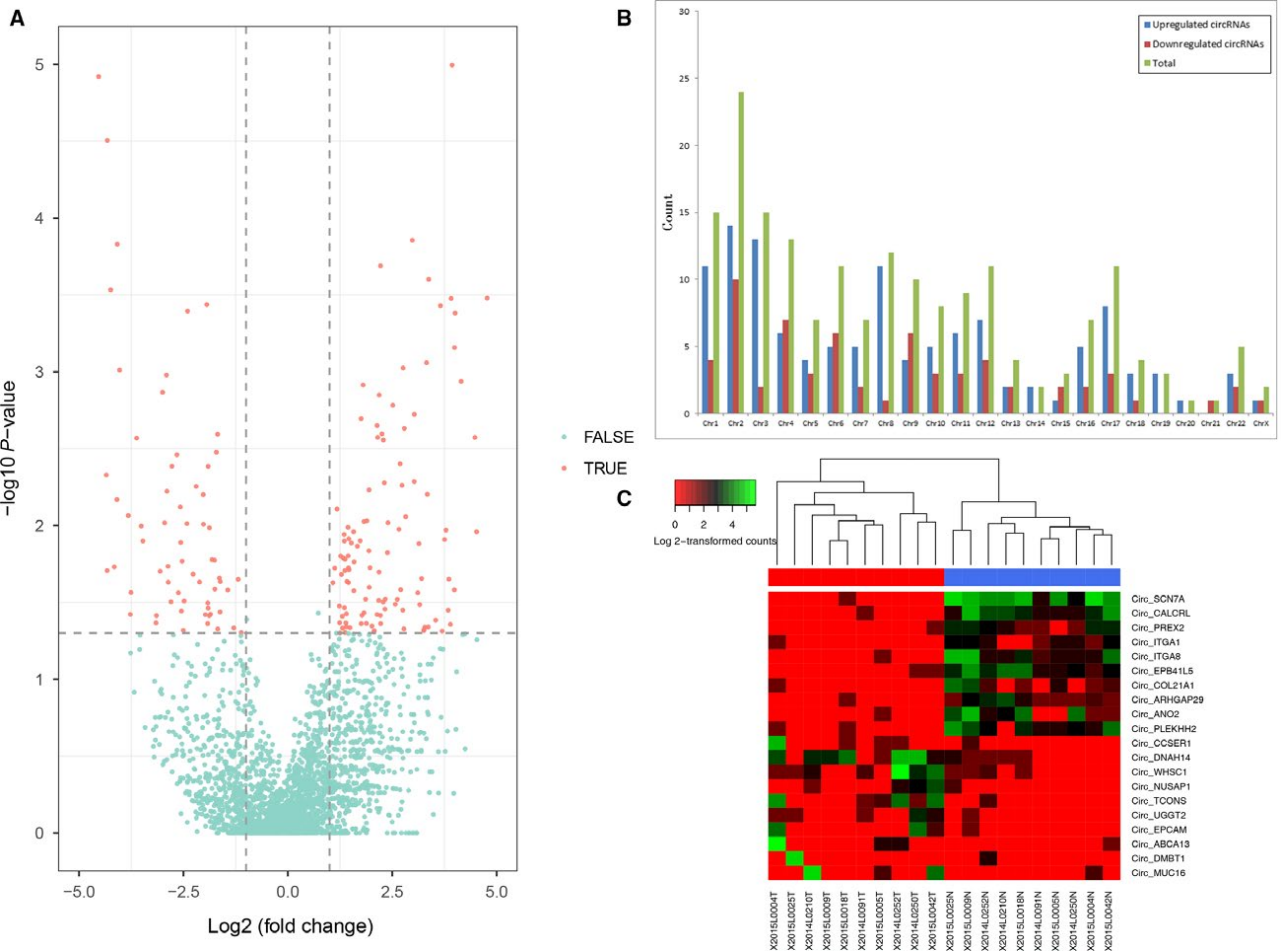
Differences in circRNA expression profiles between NSCLC and adjacent normal tissues. (A) Volcano plots showing differential expression of circRNAs between the two groups. The red points represent the differentially expressed circRNAs with fold change ≥2.0 (log2 scaled) and *P *<* *0.05 (−log10 scaled). (B) The distribution of differentially expressed circRNAs in human chromosomes. (C) Hierarchical clustering analysis of the top 10 upregulated and downregulated circRNAs.

A total of 5471 circRNAs were detected by RNA‐seq in 10 pairs of samples. The volcano plot filtering showed differentially expressed circRNAs between NSCLC and adjacent normal tissues (Fig. 1A). We found that 185 circRNAs were differentially regulated by fold change ≥2.0 (*P *<* *0.05), among which 120 circRNAs were upregulated while 65 circRNAs were downregulated. The top 10 upregulated and downregulated circRNAs ranked by *P* values are shown in Table S2, and the distribution of circRNAs on human chromosomes is depicted in Figure 1B. Hierarchical clustering showed that circRNA expression patterns were distinguishable between cancer and normal tissues (Fig. 1C). These data suggested that the expression of circRNAs in NSCLC tissues was different from that in adjacent normal tissues.

### Detection of candidate circRNAs in plasma from 10 patients with NSCLC

To prioritize candidate circRNAs for detection in circulation, we combined the published data of serum circRNA profile with our findings of upregulated circRNAs 17. The top 10 serum‐detectable and upregulated circRNAs are listed in Table S3 (*P *<* *0.05).

**Figure 2:**
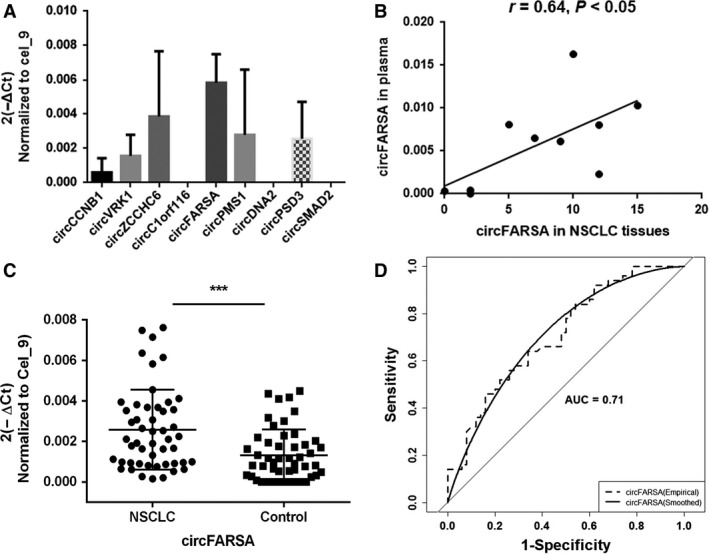
Detection of candidate circRNAs in plasma from 10 patients with NSCLC. (A) The levels of nine candidate circRNAs determined by qRT‐PCR with divergent primers. (B) The correlation of circFARSA expression between cancerous tissues and corresponding plasma. (C) The higher expression level of plasma circFARSA in NSCLC cases than that in healthy controls. Data are shown as mean ± SD, ***P <* *0.001. (D) The area under the curve by ROC analysis of plasma circFARSA.

We then determined the abundance of candidate circRNAs in plasma from the 10 patients with NSCLC who were included in RNA‐seq analysis. Except circCCDC134 due to the failure of specific primer design, the other nine circRNAs were detected in plasma by qRT‐PCR, and circFARSA exhibited the highest abundance (Fig. 2A). The level of circFARSA in plasma was correlated with its expression in cancerous tissues (*r *= 0.64, *P *=* *0.047) (Fig. 2B).

This was the first time that divergent primers of circFARSA were designed, and C. elegans cel_9 was spiked as an internal control, without primer dimers or nonspecific amplification (Fig. S2). By sequencing the amplified product of circFARSA, we confirmed the sequences consistent with that from CircBase (http://www.circbase.org) (Fig. S3).

### Upregulated expression of plasma circFARSA in patients with NSCLC

The level of plasma circFARSA was determined in 50 NSCLC cases and 50 healthy controls by qRT‐PCR (Fig. 2C). We found that circFARSA expression was higher in NSCLC cases than that in the controls (*P *<* *0.001). We also tested the level of FARSA mRNA in plasma and found that its abundance was too low to be detected.

We further analyzed the relationship between plasma circFARSA and clinicopathological features of patients (Table S4). No statistically significant association was observed, except for gender (*P *=* *0.048).

The ROC curve was plotted to evaluate the diagnostic value of circFARSA in distinguishing patients with NSCLC from cancer‐free individuals (Fig. 2D). The area under the ROC curve was 0.71.

### Functional phenotype of circFARSA

**Figure 3:**
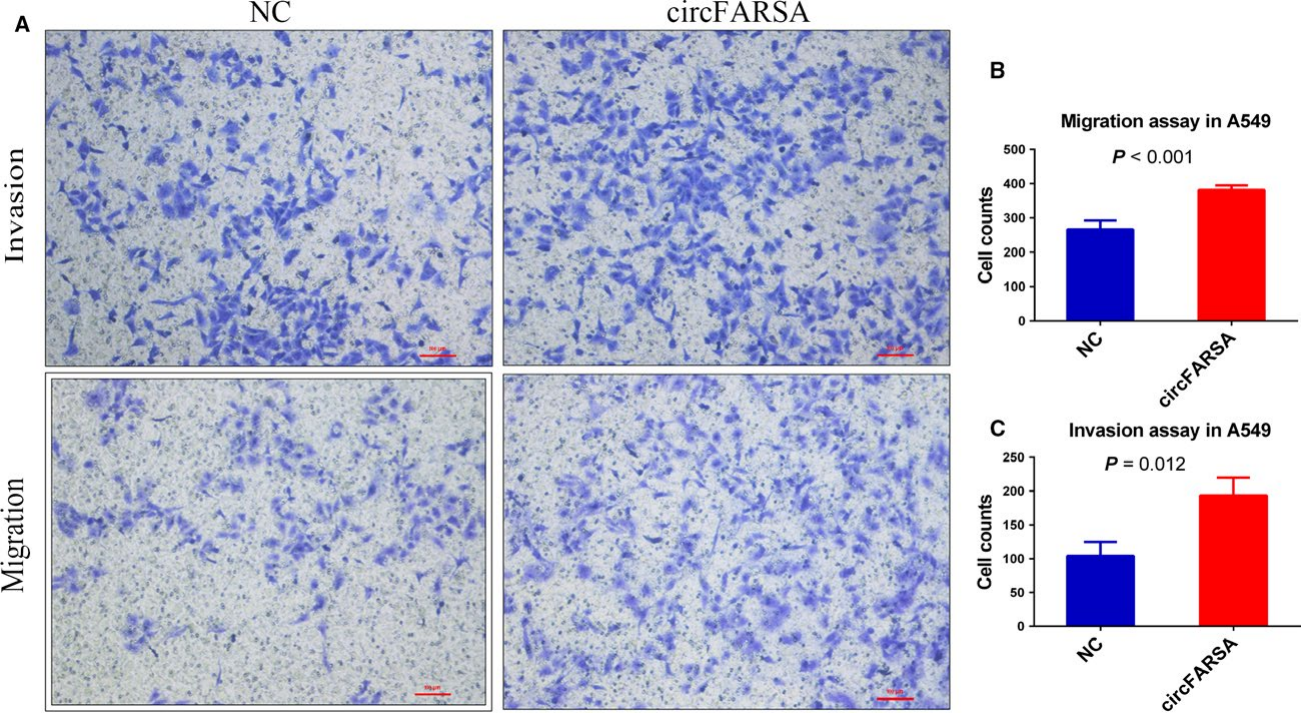
Functional assays of migration and invasion for circFARSA. (A) Migration and invasion assays of A549 cells after transfection with or without circFARSA plasmid. (B) Histogram of cell count in migration assay. (C) Histogram of cell count in invasion assay.

Overexpression of circFARSA in transfected A549 cells was confirmed by qRT‐PCR (Fig. S4A). Compared to cells transfected with empty vector, cells with circFARSA plasmid exhibited similar ability of proliferation (Fig. S5), but higher abilities of migration and invasion (Fig. 3). To exclude the possibility that increased migration and invasion characteristics of A549 cells were caused by FARSA mRNA, we detected FARSA mRNA levels in A549 cells transfected with or without circFARSA plasmid. We found that FARSA mRNA was not changed by plasmid transfection (Fig. S4B).

### CircFARSA‐targeted miRNA–mRNA pathway

**Figure 4:**
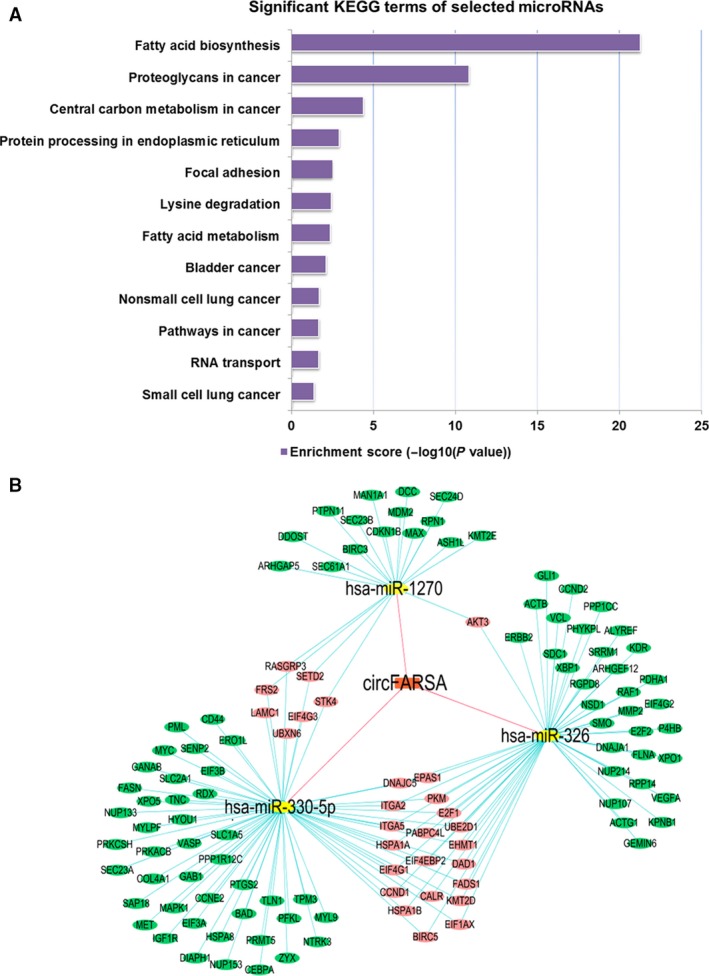
In silico analysis of the circFARSA–miRNA–mRNA interaction. (A) KEGG pathway analysis based on the circFARSA–miRNA network. Significantly enriched biological processes and their scores are listed. (B) Cytoscape analysis of circFARSA–miRNA–mRNAs network. CircFARSA‐targeted microRNAs were predicted by Circular RNA Interactome, and microRNA‐targeted mRNAs were analyzed by DIANA‐TarBase v.7.0.

Using Circular RNA Interactome, the top five miRNAs targeted by circFARSA were identified as follows: miR‐330‐5p, miR‐326, miR‐1178‐3p, miR‐620, and miR‐1270. Based on KEGG annotation, 12 pathways were identified and fatty acid biosynthesis was the most statistically significant pathway (Fig. 4A). Cytoscape analysis of circRNA–microRNA–mRNA interaction found that miR‐330‐5p and miR‐326 exhibited the largest interaction network (Fig. 4B).

According to HITS‐CLIP experiment evidence from TarBase, both miR‐330‐5p and miR‐326 may directly interact with fatty acid synthase (FASN) which emerges as an oncogene in various cancers. In TCGA datasets, FASN expression was increased in both lung adenocarcinoma (*n *= 57, *P *<* *0.001) and squamous carcinoma (*n *= 50, *P *=* *0.005), compared with paired normal tissues (Fig. S6A). Moreover, microarray data from Kaplan–Meier plotter (*n *= 1926) indicated the prognostic role of FASN expression in lung cancer patients (HR = 1.81, 95% CI: 1.47–2.21, adjusted for clinical stage) (Fig. S6B).

## Discussion

In the present study, we utilized RNA‐seq and revealed 120 upregulated circRNAs and 65 downregulated circRNAs in NSCLC tissues. To prioritize candidate circRNAs for detection in plasma, we integrated the published data of serum circRNA profile with our findings. Then, we demonstrated that circFARSA exhibited the highest abundance among nine serum‐detectable circRNAs. In addition, plasma circFARSA was higher in patients with NSCLC than in healthy controls. The area under the ROC curve was 0.71, suggesting that plasma circFARSA might represent a potential biomarker for NSCLC.

Recent studies suggest that specific circRNAs are abundant and stable in saliva 25, plasma/serum 17, 26, and blood 27. There is an intriguing possibility of circRNA excretion into the extracellular space by vesicles such as exosomes, which is a direct consequence of active or passive release of circRNAs from diseased tissue. Li et al. showed that serum exosome‐circRNAs were able to distinguish patients with colorectal cancer from healthy controls 17. In the present study, we found a correlation between the levels of circFARSA in NSCLC tissues and plasma. However, by comparing circFARSA in plasma with that in exosomes extracted from the same patients’ equal amount of plasma, we found that the abundance of circFARSA was higher in plasma (data not shown). We speculate that other ways might exist for the release of circFARSA into the circulation.

CircRNAs have been demonstrated to act as miRNA sponges and regulate the expression of miRNA‐targeted transcripts 6, 9. For instance, circRNA CDR1as could bind to miR‐7 and downregulated a number of oncogenes such as EGFR, and circHIPK3 sequestered multiple miRNAs, including a well‐known tumor suppressor miR‐124 14. In the present study, we observed that overexpressed circFARSA promoted cell migration and invasion in vitro. Bioinformatics analysis suggested that circFARSA contained conserved seed matches to miR‐330‐5p, miR‐326, miR‐1178‐3p, miR‐620, and miR‐1270. These miRNAs may be implicated in several cancer‐related pathways, including fatty acid biosynthesis that is critical for cell proliferation and metastasis. Previous studies showed that miR‐330‐5p inhibited oncogene MUC1 expression in pancreatic cancer cells and downregulated ITGA5 expression in colorectal cancer 28, 29. In addition, miR‐326 could inhibit malignant phenotypes of NSCLC through downregulation of oncogene CCND1 30, 31, 32. According to TarBase, both miR‐330‐5p and miR‐326 might directly interact with FASN, a metabolic oncogene in different types of cancer 33. Moreover, by analyzing TCGA datasets and online microarray data, we confirmed the overexpression of FASN in lung cancer and its association with poor prognosis. Therefore, our findings supported the hypothesis that circFARSA might contribute to the development of lung cancer by sponging miR‐330‐5p/miR‐326 and relieving their inhibitory effects on oncogene FASN.

The present study has several strengths. First, using high‐throughput RNA‐seq, we provided a global insight of differently expressed circRNAs in NSCLC. Second, it is the first attempt to explore the potential of plasma circRNAs as novel biomarkers for patients with NSCLC. Third, we demonstrated that a C. elegan circRNA, cel_9, was stable in human plasma and suitable as internal reference. Finally, both in silico and in vitro evidences suggested the crucial role of circFARSA in lung cancer progression. However, there are also several limitations. First, although we conducted two‐stage design with the screening and validation stages, the sample size was relatively small. Other circRNAs in the profile might also have the potential as lung cancer biomarkers. Future studies with large sample size in the first tissue‐screening stage and second plasma‐validation stage are warranted. Second, additional in‐depth functional studies are essential to unravel molecular mechanism of circFARSA–miRNA–FASN interactions in NSCLC.

In conclusion, the present study provides the precursory evidence to foster the potential of plasma circFARSA as a novel biomarker for NSCLC. Our findings would shed light on future follow‐up studies on circulating circRNAs as noninvasive biomarkers for cancer.

## Conflict of Interest

The authors have no conflict of interest to disclose.

## Supporting information


**Figure S1.** Flow chart of circRNA selection.
**Figure S2.** Specific melting peaks for qRT‐PCR products of circFARSA and cel_9 in plasma (A)circFARSA; (B)cel_9.
**Figure S3.** Sequencing result of qRT‐PCR product and the schematic diagram of circFARSA.
**Figure S4.** (A)The expression levels of circFARSA in A549 cells after transfection with or without circFARSA plasmid. Data are shown as mean ± SD, ***P <* *0.01, *n *= 3; (B) The expression levels of FARSA mRNA in A549 cells after transfection with or without circFARSA plasmid. Data are shown as mean ± SD, ns: *P* value not significant, *n *= 3.
**Figure S5.** CCK8 cell proliferation assay of A549 cells after transfection with or without circFARSA plasmid.
**Figure S6.** FASN expression in lung cancer and its association with prognosis.

**Table S1.** Clinicopathological features of 10 NSCLC patients in RNA‐seq analysis.
**Table S2.** Top 10 up‐regulated and down‐regulated circRNAs between NSCLC cancerous and adjacent normal tissues.
**Table S3.** Top 10 candidate circRNAs in plasma from NSCLC patients.
**Table S4.** The relationship between plasma circFARSA and clinicopathological features of NSCLC patients.
