# Supplementary material for: A novel plasma circular RNA circFARSA is a potential biomarker for non‐small cell lung cancer
Source: Cancer Med. 2018 May 2;7(6):2783–91. doi: 10.1002/cam4.1514 (PMC6010816; doi:10.1002/cam4.1514)
Supplement: Supplementary file 1 — Figure S1. Flow chart of circRNA selection. Figure S2. Specific melting peaks for qRT‐PCR products of circFARSA and cel_9 in plasma (A)circFARSA; (B)cel_9. Figure S3. Sequencing result of qRT‐PCR product and the schematic diagram of circFARSA. Figure S4. (A)The expression levels of circFARSA in A549 cells after transfection with or without circFARSA plasmid. Data are shown as mean ± SD, ***P < 0.01, n = 3; (B) The expression levels of FARSA mRNA in A549 cells after transfection with or without circFARSA plasmid. Data are shown as mean ± SD, ns: P value not significant, n = 3. Figure S5. CCK8 cell proliferation assay of A549 cells after transfection with or without circFARSA plasmid. Figure S6. FASN expression in lung cancer and its association with prognosis. [file CAM4-7-2783-s001.pdf]

Supplementary Figures

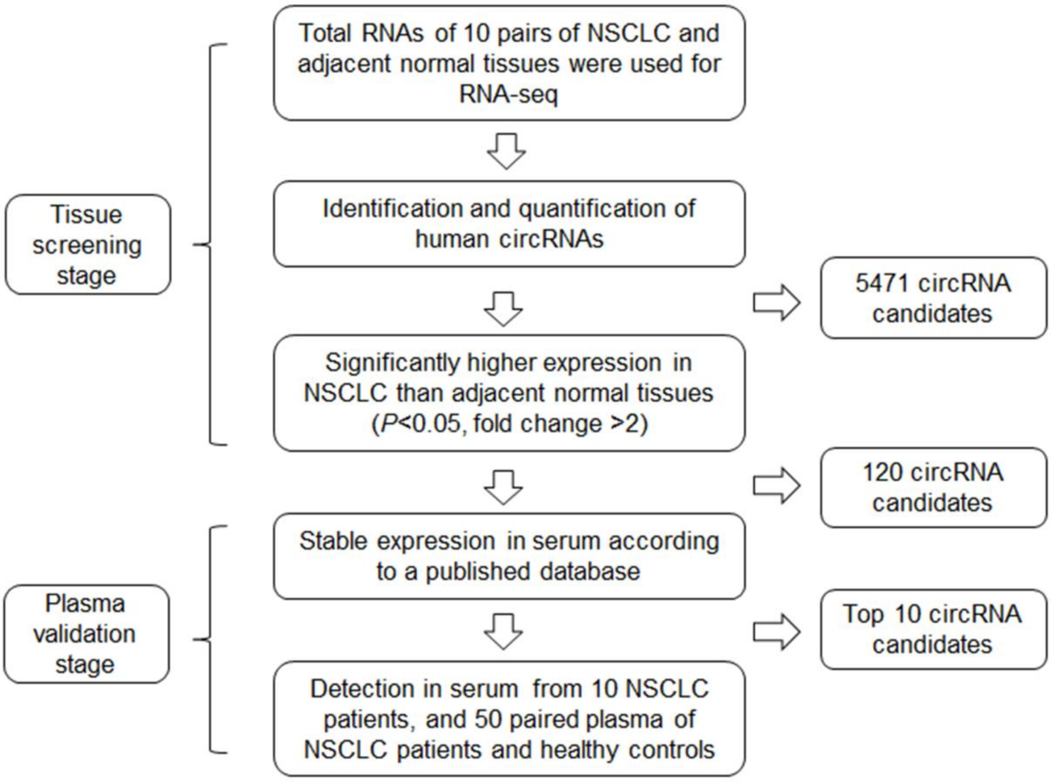

Figure S1. Flow chart of circRNA selection.

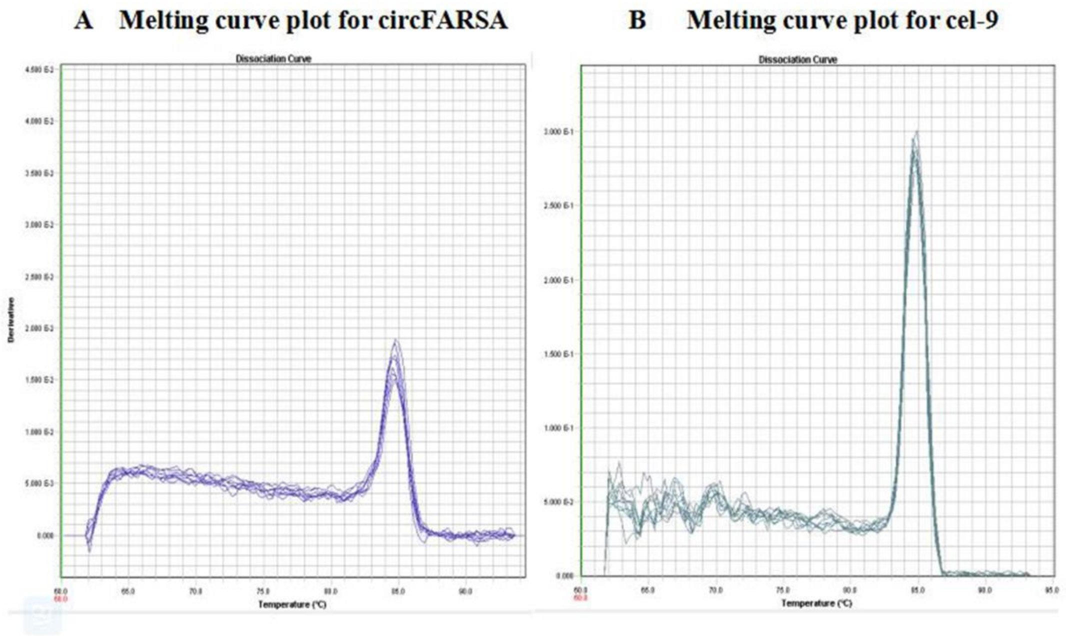

Figure S2. Specific melting peaks for qRT-PCR products of circFARSA (A) and cel_9 (B) in plasma.

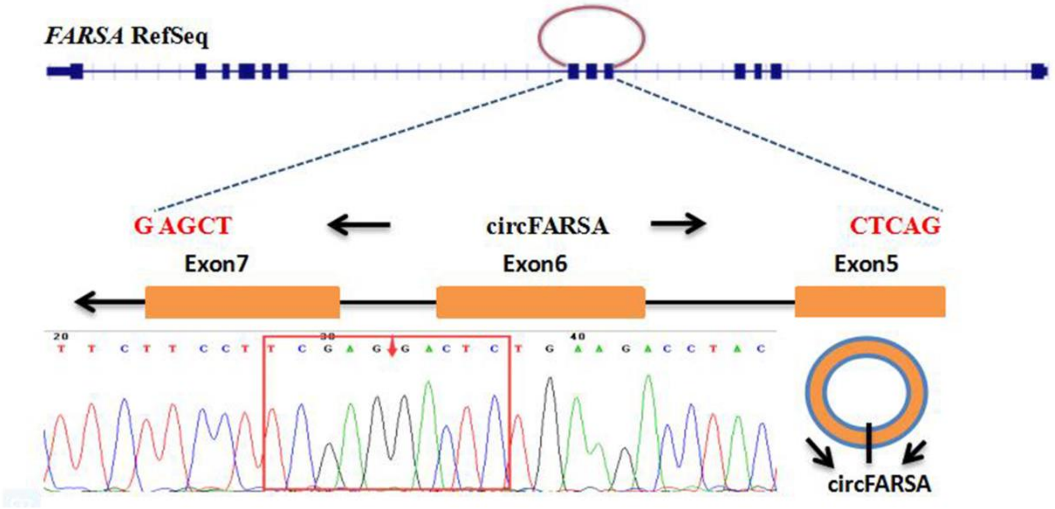

Figure S3. Sequencing result of qRT-PCR product and the schematic diagram of circFARSA.

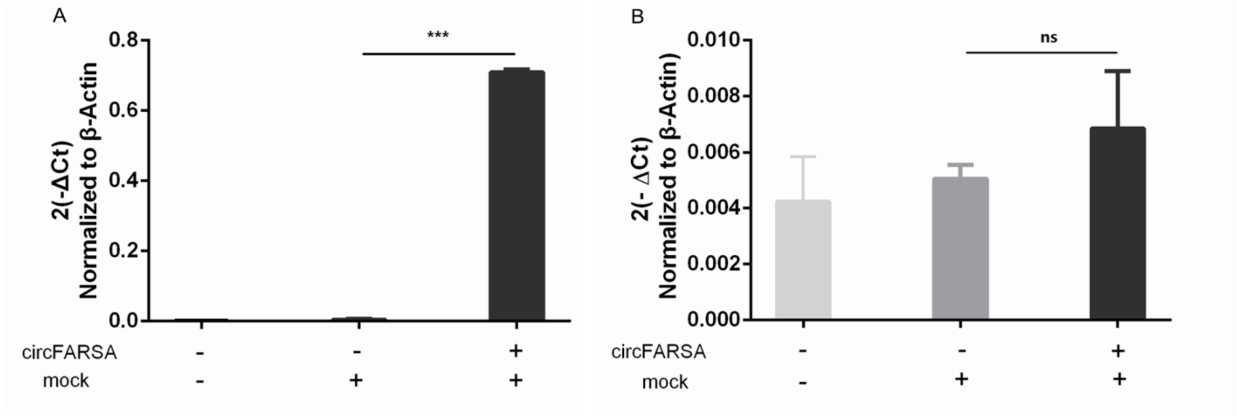

Figure S4. (A)The expression levels of circFARSA in A549 cells after transfection with or without circFARSA plasmid. Data are shown as mean ± SD, ***P < 0.01, n=3; (B) The expression levels of FARSA mRNA in A549 cells after transfection with or without circFARSA plasmid. Data are shown as mean ± SD, ns: P value not significant, n=3.

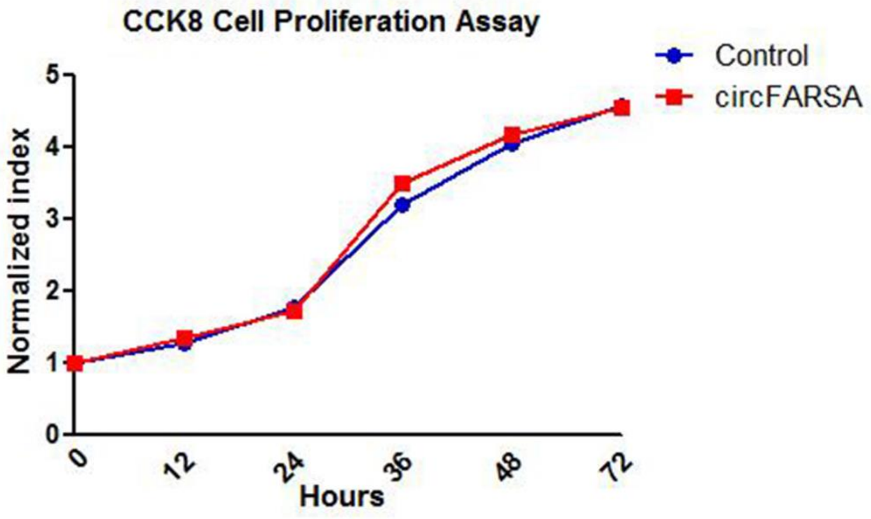

Figure S5. CCK8 cell proliferation assay of A549 cells after transfection with or without circFARSA plasmid.

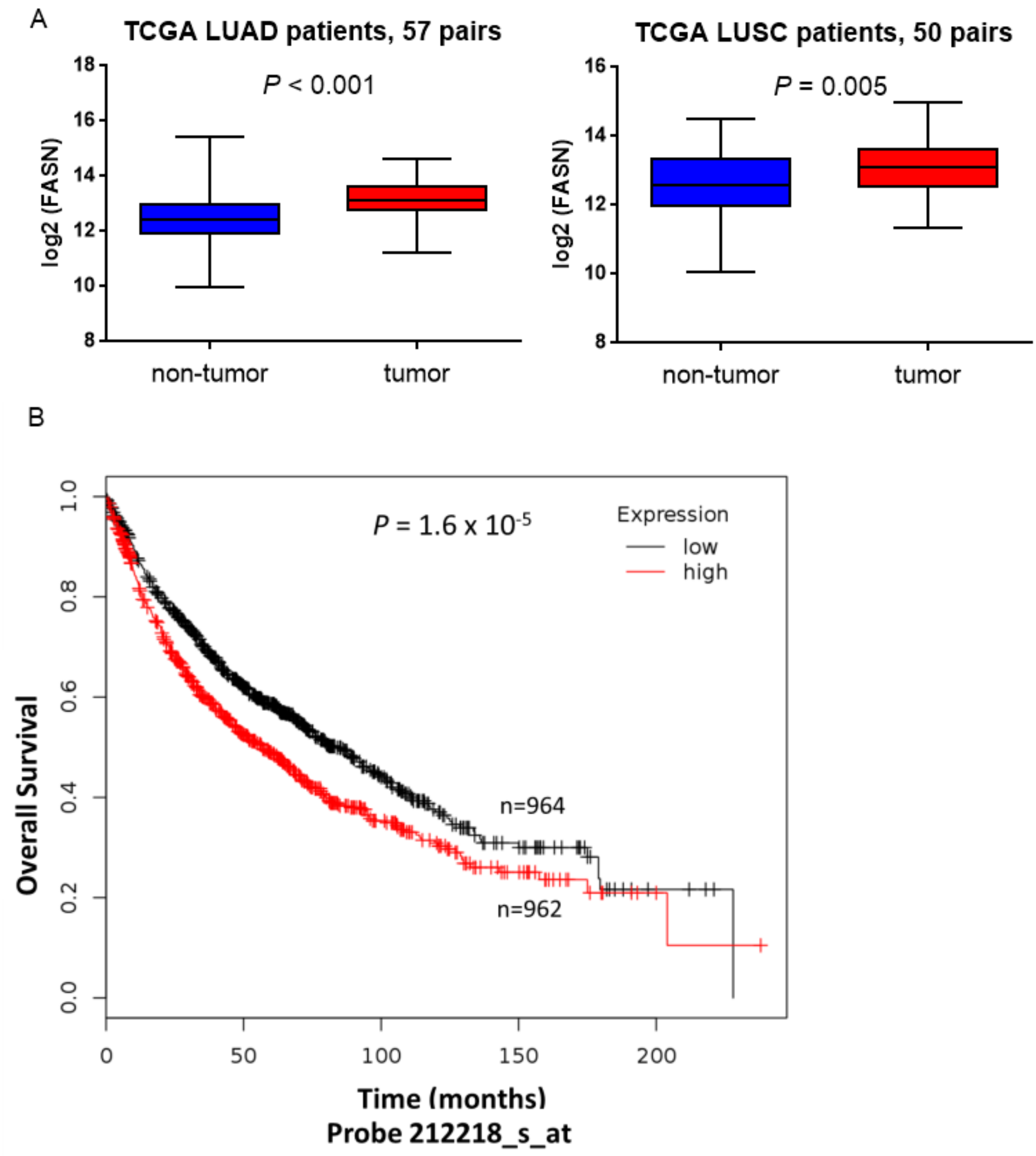

Figure S6. FASN expression in lung cancer and its association with prognosis. (A) TCGA LUAD and LUAC datasets showing the higher expression of FASN in cancerous tissues than in adjacent normal tissues. (B) Kaplan–Meier curves of overall survival according to FASN expression (probe 212218_s_at) in 1926 lung cancer patients using microarray data from Kaplan-Meier plotter (http://www.kmplot.com/lung).
