# Supplementary material for: A novel plasma circular RNA circFARSA is a potential biomarker for non‐small cell lung cancer
Source: Cancer Med. 2018 May 2;7(6):2783–91. doi: 10.1002/cam4.1514 (PMC6010816; doi:10.1002/cam4.1514)
Supplement: Supplementary file 2 — Table S1. Clinicopathological features of 10 NSCLC patients in RNA‐seq analysis. Table S2. Top 10 up‐regulated and down‐regulated circRNAs between NSCLC cancerous and adjacent normal tissues. Table S3. Top 10 candidate circRNAs in plasma from NSCLC patients. Table S4. The relationship between plasma circFARSA and clinicopathological features of NSCLC patients. [file CAM4-7-2783-s002.docx]

**Supplementary Tables**

| **Table S1.** Clinicopathological features of 10 NSCLC patients in RNA-seq analysis | |
| --- | --- |
| Characteristics | No. of patients |
| Age (year) |  |
| ≤60 | 5 |
| >60 | 5 |
| Gender |  |
| Male | 8 |
| Female | 2 |
| Smoking |  |
| Ever | 7 |
| Never | 3 |
| Differentiation |  |
| Moderate | 3 |
| Poor | 7 |
| Histologic type |  |
| Squamous carcinoma | 4 |
| Adenocarcinoma | 6 |
| TNM stage |  |
| I | 5 |
| II | 5 |

| **Table S2.** Top 10 up-regulated and down-regulated circRNAs between NSCLC cancerous and adjacent normal tissues | | | | | | |  |
| --- | --- | --- | --- | --- | --- | --- | --- |
| Gene | circRNA | CirBase ID | Region | Annotation | Fold change^a^ | *P*^b^ | |
| TCONS | circ_TCONS | hsa_circ_0001944 | chrX:130883333-130928494 | intergenic | 22.4 | 5.67E-07 | |
| WHSCI | circ_WHSC1 | hsa_circ_0004156 | chr4: 1902352-1920350 | exonic | 19.7 | 7.56E-07 | |
| DNAH14 | circ_DNAH14 | hsa_circ_0016599 | chr1: 225140371-225156576 | exonic | 18.5 | 8.70E-07 | |
| MUC16 | circ_MUC16 | hsa_circ_0049119 | chr19: 9007486-9012898 | exonic | 44.5 | 9.41E-07 | |
| ABCA13 | circ_ABCA13 | NA | chr7: 48443279-48467468 | exonic | 27.7 | 3.20E-06 | |
| DMBT1 | circ_DMBT1 | NA | chr10: 124345575-124379807 | exonic | 32.2 | 1.24E-04 | |
| EPCAM | circ_EPCAM | NA | chr2: 47600601-47601187 | exonic | 27.3 | 3.32E-04 | |
| CCSER1 | circ_CCSER1 | NA | chr4: 91229394-91234198 | exonic | 17.8 | 1.15E-03 | |
| NUSAP1 | circ_NUSAP1 | hsa_circ_0034693 | chr15: 41648236-41650456 | exonic | 22.3 | 2.67E-03 | |
| UGGT2 | circ_UGGT2 | hsa_circ_0008274 | chr13: 96485180-96489456 | exonic | 22.9 | 1.10E-02 | |

NA, not available.

^a^ The expression level in cancerous tissues were compared with that in adjacent normal tissues.

^b^ Derived from the DEseq2 method.

| **Table S3.** Top 10 candidate circRNAs in plasma from NSCLC patients | | | | | | | | |
| --- | --- | --- | --- | --- | --- | --- | --- | --- |
| Gene | circRNA | CirBase ID | Serum ID | Region | Size (bp) | Annotation | Fold change^a^ | *P*^b^ |
| CCNB1 | circCCNB1 | has_circ_0001495 | Serum_circ_0916 | chr5: 68470703-68471364 | 378 | CDS/Exon | 8.2 | 0.002 |
| VRK1 | cirVRK1 | hsa_circ_0000566 | Serum_circ_0380 | chr14: 97299803-97327072 | 1073 | CDS/Exon/5'UTR | 4.9 | 0.003 |
| CCDC134 | circCCDC134 | hsa_circ_0001238 | Serum_circ_0744 | chr22: 42204878-42206295 | 326 | CDS/Exon/5'UTR | 3.0 | 0.011 |
| ZCCHC6 | circZCCHC6 | hsa_circ_0007037 | Serum_circ_1166 | chr9: 88918000-88919862 | 208 | CDS/Exon | 2.8 | 0.012 |
| C1orf116 | circC1orf116 | NA | Serum_circ_0104 | chr1: 207200838-207201024 | 186 | CDS/Exon/5'UTR | 2.9 | 0.013 |
| FARSA | circFARSA | hsa_circ_0000896 | Serum_circ_0532 | chr19:13039155-13039661 | 338 | CDS/Exon | 2.7 | 0.016 |
| PMS1 | circPMS1 | hsa_circ_0001083 | Serum_circ_0654 | chr2: 190656515-190682906 | 602 | CDS/Exon/5'UTR | 4.5 | 0.031 |
| DNA2 | circDNA2 | hsa_circ_0006151 | Serum_circ_0160 | chr10:70227879-70229920 | 367 | CDS/Exon/3'UTR | 3.4 | 0.043 |
| PSD3 | circPSD3 | hsa_circ_0004458 | Serum_circ_1080 | chr8:18656804-18662408 | 448 | CDS/Exon | 2.4 | 0.043 |
| SMAD2 | circSMAD2 | hsa_circ_0000847 | Serum_circ_0512 | chr18:45391429-45423180 | 783 | CDS/Exon/5'UTR | 4.0 | 0.045 |

NA, not available.

^a^ The expression level in cancerous tissues were compared with that in adjacent normal tissues.

^b^ Derived from the DEseq2 method.

**Table S4.** The relationship between plasma circFARSA and clinicopathological features of NSCLC patients

| Characteristics | No. of patients (%) | Mean ± SD | *P* ^a^ |
| --- | --- | --- | --- |
| Age (year) |  |  | 0.684 |
| ≤60 | 18 (36%) | 2.78 × 10^-3^ ± 2.18 × 10^-3^ |  |
| >60 | 32 (64%) | 2.47 × 10^-3^ ± 1.88 × 10^-3^ |  |
| Gender |  |  | 0.048 |
| Male | 33 (66%) | 2.24 × 10^-3^ ± 1.83 × 10^-3^ |  |
| Female | 17 (34%) | 3.25 × 10^-3^ ± 2.13 × 10^-3^ |  |
| Smoking |  |  | 0.476 |
| Current | 18 (36%) | 2.51 × 10^-3^ ± 2.02 × 10^-3^ |  |
| Ever | 6 (12%) | 1.77 × 10^-3^ ± 1.28 × 10^-3^ |  |
| Never | 26 (52%) | 2.82 × 10^-3^ ± 2.08 × 10^-3^ |  |
| Differentiation |  |  | 0.131 |
| Well/Moderate | 35 (70%) | 2.98 × 10^-3^ ± 2.16 × 10^-3^ |  |
| Poor | 15 (30%) | 2.95 × 10^-3^ ± 2.61 × 10^-3^ |  |
| Histologic type |  |  | 0.270 |
| Squamous carcinoma | 21 (42%) | 2.36 × 10^-3^ ± 1.94 × 10^-3^ |  |
| Adenocarcinoma | 29 (58%) | 2.75 × 10^-3^ ± 2.02 × 10^-3^ |  |
| TNM stage |  |  | 0.678 |
| I-II | 28 (56%) | 2.70 × 10^-3^ ± 2.11 × 10^-3^ |  |
| III-IV | 22 (44%) | 2.43 × 10^-3^ ± 1.83 × 10^-3^ |  |

^a^ Paired t-test or one-way analysis was performed using the log-transformed data.
